# Paediatric Patella Tendon Rupture: A Systematic Review

**DOI:** 10.7759/cureus.115603

**Published:** 2026-09-01

**Authors:** Abu-Bakr I Ashfaq, Amaar A Ali

**Affiliations:** 1 Trauma and Orthopaedics, University College London, London, GBR; 2 Trauma and Orthopaedics, Royal Lancaster Infirmary, Lancaster, GBR

**Keywords:** adolescent, children, knee injuries, patella tendon, sport injury

## Abstract

The patella tendon is an important part of the extensor mechanism, and ruptures can lead to significant impairment. The condition is rare in children and adolescents, and as a result, the paediatric literature is limited. Injury patterns may differ from those seen in adults. This systematic review therefore aims to evaluate the presentation, diagnosis, management and outcomes of patella tendon ruptures in children and adolescents. A literature search was performed using PubMed, Medline and Embase to identify studies based on the Preferred Reporting Items for Systematic Reviews and Meta-analyses (PRISMA) guidelines. The search period included all studies up until the 22nd May 2026. A total of 350 studies were identified from the initial literature search after duplicates were removed. Five studies covering 28 patients met the eligibility criteria and were included. Over 95% of patients with a patellar tendon injury were male. Basketball was the most common sport where ruptures occurred, and axial loading was the most commonly reported mechanism of injury. Distal patellar tendon ruptures were more common than proximal or mid-substance tears. Over 70% of cases had an associated tibial tubercle fracture, although this was affected by selection bias since 3 of the 5 studies specifically enrolled patients with tibial tubercle fractures. Three studies noted patella alta, or a high-rising patella, in all 10 documented cases of injury in which it was assessed. All patients were managed surgically predominantly via the use of transosseous tunnels or suture anchors. Reported outcomes were generally favourable and included active range of motion, the ability to weight bear, and quadriceps strength. To conclude, paediatric patella tendon ruptures are uncommon but should be considered in children presenting with acute knee trauma and extensor mechanism dysfunction, particularly where tibial tubercle fractures are diagnosed. Larger multi-centre studies with standardised diagnostic, operative and outcome reporting are needed in future research.

## Introduction and background

Patellar tendon rupture is an uncommon but clinically important injury involving disruption of the knee extensor mechanism. Together with the quadriceps muscles, quadriceps tendon and patella, the patellar tendon allows active knee extension and plays an essential role in walking and many other activities [1,2]. Loss of continuity of the extensor mechanism can therefore cause marked functional impairment, particularly through weakness or loss of active knee extension.

Patellar tendon ruptures are rare compared with many other knee injuries. In adults, reported incidence is around 0.68 per 100,000 person-years, although rates are higher in more active groups [3,4]. It is most often described in males and is commonly associated with sporting activity, especially in younger adults.

The typical mechanism involves sudden overload of the extensor mechanism, usually when a forceful quadriceps contraction occurs against a flexed knee. This can occur during jumping, sudden deceleration or a fall onto a flexed knee [1,5]. In adults, ruptures are often associated with pre-existing tendon degeneration or systemic factors including chronic tendinopathy, inflammatory disease, renal disease, diabetes, and corticosteroid and fluoroquinolone use [5,6].

In adults, complete patellar tendon rupture is usually managed surgically as restoration of the extensor mechanism is required to regain active knee extension. Active repair aims to restore tendon continuity and patellar height. Techniques vary according to the rupture site, but include end-to-end sutures for mid-substance tears, or tendon-to-bone fixation using transosseous tunnels or suture anchors for avulsion-type injuries [7,8]. Delayed or insufficient treatment can lead to extensor lag, persistent weakness and stiffness, a reduced range of motion and re-rupturing [1,5,7]. 

The paediatric knee contains open physes and apophyseal attachment sites, meaning that force transmitted through the extensor mechanism may produce different patterns of injury. Rather than a pure tendon rupture, children may sustain bony, osteochondral or apophyseal avulsion injuries [2,9]. The paediatric extensor mechanism is therefore affected by a distinct spectrum of injuries related to skeletal immaturity. Consequently, whilst ruptures in the general population usually occur midsubstance as pure tendon ruptures, in children, they may be more likely to occur at the tendon insertions as avulsion injuries. Another explanation could potentially be that patellar tendons are longer and stiffer in adults than children [10]. This can result in greater stress within the tendon midsubstance as opposed to the surrounding tissue.

Despite these important differences, paediatric patellar tendon ruptures remain poorly represented in the literature. Existing evidence is limited by the rarity of the condition. A systematic review is needed to summarise the available evidence and clarify how patellar tendon injuries present and are managed in the paediatric population. This review aims to evaluate the published literature on patellar tendon ruptures in children and adolescents, with a focus on injury patterns, mechanisms of injury, clinical presentation, management strategies and reported outcomes.

## Review

Methods

This systematic review was performed in line with the PRISMA guidelines [11] and was not prospectively registered. 

Search Strategy

Table 1 depicts the full search strategy, which was developed upon consultation of two reviewers: AA and AA. The same search term was applied to ‘Title and Abstract’ fields in all databases searched. The search period encompassed all studies published up until the 22nd May 2026. 

**Table 1:** Search strategy

Search term 1	Search term 2	Search term 3
patella* tendon	tear*	child*
patella* ligament	torn	paediatric
infrapatella* tendon	ruptur*	pediatric
infrapatella* ligament	injur*	adolescent*
ligamentum patellae	break*	teen*
	avulsion	juvenile
	discontinuity	
	laceration*	
	detach*	
	separat*	
	snap*	

Database Search Strategy

Below are the specific search strategies for each database searched (Table 2).

**Table 2:** Search strategy with search strings for each database

Database	Search strategy	Limit
PubMed	Search Title/Abstract: (("Patella* tendon"[Title/Abstract] OR "patella* ligament"[Title/Abstract] OR "infrapatella* tendon"[Title/Abstract] OR "infrapatella* ligament"[Title/Abstract] OR "ligamentum patellae"[Title/Abstract]) AND (Tear*[Title/Abstract] OR torn[Title/Abstract] OR ruptur*[Title/Abstract] OR injur*[Title/Abstract] OR break[Title/Abstract] OR avulsion[Title/Abstract] OR discontinuity[Title/Abstract] OR laceration*[Title/Abstract] OR detach*[Title/Abstract] OR separat*[Title/Abstract] OR snap[Title/Abstract])) AND (Child*[Title/Abstract] OR paediatric[Title/Abstract] OR paediatric[Title/Abstract] OR adolescent*[Title/Abstract] OR teen*[Title/Abstract] OR juvenile[Title/Abstract])	—
MEDLINE	Search Title: (("Patella* tendon" or "patella* ligament" or "infrapatella* tendon" or "infrapatella* ligament" or "ligamentum patellae") and (Tear* or torn or ruptur* or injur* or break or avulsion or discontinuity or laceration* or detach* or separat* or snap*)).mp. and (Child* or paediatric or paediatric or adolescent* or teen* OR juvenile).m_titl.	Limit to abstracts
Embase	Search Title: (("Patella* tendon" or "patella* ligament" or "infrapatella* tendon" or "infrapatella* ligament" or "ligamentum patellae") and (Tear* or torn or ruptur* or injur* or break or avulsion or discontinuity or laceration* or detach* or separat* or snap*)).mp. and (Child* or paediatric or paediatric or adolescent* or teen* or juvenile).m_titl.	Limit to abstracts

Screening and Data Extraction

Three major databases were searched: PubMed, Medline and Embase, which were felt to provide sufficient breadth to capture the relevant evidence base. The retrieved papers were imported into EndNote, and duplicates were removed. Titles and abstracts were screened using the inclusion and exclusion criteria outlined in Table 3. According to the WHO definition, the upper limit of adolescence has been taken as 19 years old [12]. ‘Full Text’ screening was then undertaken to select included studies. Screening and data extraction were carried out by each of the 2 reviewers independently. Any disagreements were resolved upon consultation during a planned meeting.

**Table 3:** Inclusion and exclusion criteria

	Inclusion	Exclusion
Population	Children and adolescents aged 19 or younger with a rupture of the patella tendon	Adult patients
Intervention	-	-
Comparison	-	-
Outcome	Causes and mechanisms of injury, diagnosis, management and any recorded outcomes of patella tendon ruptures	Causes and mechanisms of injury, diagnosis, management and any recorded outcomes regarding other tendon or knee injuries
Study type	Original academic literature in the English language	Abstracts, grey literature, single case reports, reviews, animal studies, studies not in English

Synthesis of Results and Quality Assessment

A risk of bias assessment was conducted using the JBI Critical Appraisal Checklist for Case Series for papers that were case series, and the modified Newcastle-Ottawa scale for a paper that was a retrospective observational study [13].

The findings were presented using a narrative synthesis, with ‘Braun and Clarke’s six steps’ used to guide a thematic analysis [14]. After data extraction and familiarisation with the data, the findings were coded according to recurring clinical concepts. These related codes were then grouped into broader categories. Once they were checked for consistency across the studies, they were refined into the themes reported in the results section. This included mechanisms of injury, diagnosis and imaging, patterns of injury and injury associations, management, and outcomes and complications. A quantitative synthesis was not appropriate due to the small number of heterogeneous studies.

Results

Figure 1 shows a PRISMA diagram summarising the screening process. 588 papers were found from searching three databases. After removing 238 duplicates, title and abstract screening was conducted on 350 papers. A total of 11 papers were sought for retrieval. Two could not be retrieved due to inaccessibility, which could introduce a source of bias. The remaining nine were subsequently screened for their full text, leaving five papers that were accessible and met the inclusion and exclusion criteria. A summary of each paper is presented in Table 4.

**Figure 1:**
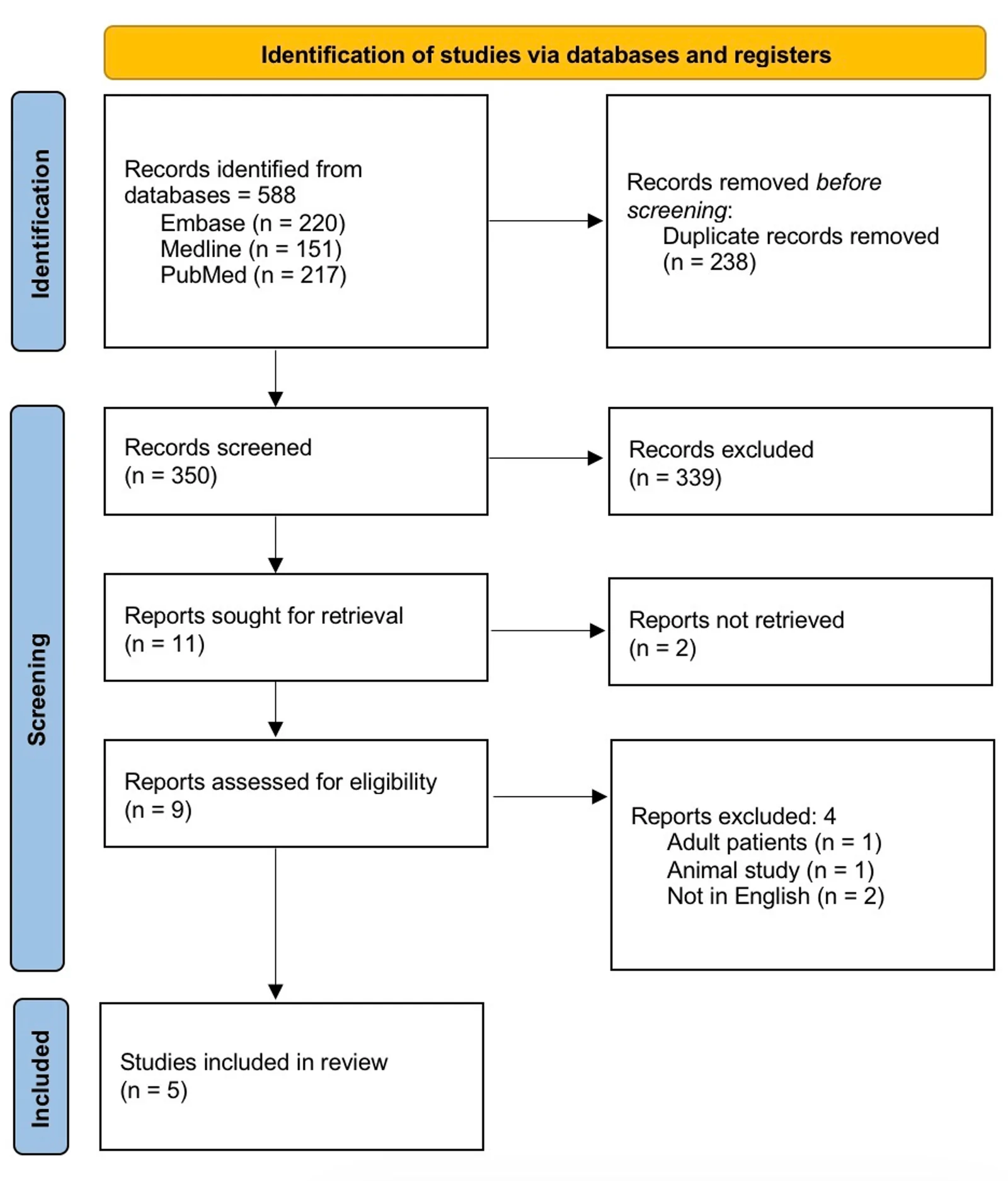
PRISMA diagram

**Table 4:** Overview of studies

Author, year	Study type	Sample size	Average age (years)	Male/female ratio	Average follow-up (months)
Ali Yousef and Rosenfeld, 2017 [15]	Retrospective case-series	5	13.6	5/0	4.2
Bradko et al., 2018 [16]	Retrospective case-series	2	12.5	2/0	3
Schultz et al., 2025 [17]	Retrospective observational	16	13.75	15/1	N/A
Wood et al., 2024 [18]	Retrospective case-series	2	14	2/0	4
Yousef MA, 2018 [19]	Retrospective case-series	3	13.7	3/0	N/A

Across the five included studies [15-19], there were 28 paediatric patients with a patellar tendon rupture. This included one study of isolated acute traumatic patellar tendon ruptures [15], three studies of combined patellar tendon avulsion with tibial tubercle fracture [16,18,19], and one larger study comparing isolated tibial tubercle fracture with combined tibial tubercle fracture and patellar tendon avulsion [17]. The mean age ranged from 12.5 to 14. Most patients were male, with 1 female and 27 male patients reported across all the studies combined. It should be acknowledged that the patient cohorts in both of Yousef’s papers are different [15,19].

Mechanism of Injury

All ruptures were traumatic in nature. The frequency of different mechanisms of injury across all five studies has been outlined in Table 5 below. Sports were the most frequent cause, of which basketball was the most common. In the larger study by Schultz et al., 89% of injuries were described as high energy [17]. Mechanisms were grouped in broad categories, with axial loading being the most common at 56%, followed by falls at 17% and collision injuries at 13% [17].

**Table 5:** Mechanism of injury

Mechanism of injury	Number of patients
Basketball	7
Axial loading	8
Falls	4
Hurdling	2
Collisions	2
Gymnastics	1
Running	1
Not specified	3

Diagnosis and Imaging

Detailed clinical presentation was reported in four studies comprising 12 patients [15,16,18,19]. In Ali Yousef and Rosenfeld, all five patients presented with swelling, painful limitation of knee motion, and inability to bear weight [15]. Clinically suspected proximal displacement of the patella and infrapatellar tenderness were present in all patients. A palpable infrapatellar gap was present in only two patients.

Bradko et al. reported inability to bear weight in one patient and inability to walk in the second [16]. Wood et al. described acute knee pain and swelling with the inability to perform a straight-leg raise in both patients [18]. All three patients in Yousef’s 2018 study presented with the inability to bear weight and a restricted range of motion [19].

Plain radiographs were used in all cases as the initial imaging investigation. Patella alta, where the patella is positioned abnormally high, was an important finding in isolated patellar tendon ruptures [20]. In Ali Yousef and Rosenfeld [15], a high-riding patella was described as a hallmark diagnostic sign in all five patients, with an Insall-Salvati ratio of 2.18. MRI was then used to confirm diagnosis in two patients and provide more detail on the pattern of injury. This included one case where there was a complete avulsion from the tibia, and another where a delamination shear injury was identified, which was consistent with a midsubstance rupture. Out of the five patients, three had a distal avulsion, one had a proximal avulsion, and one had a midsubstance disruption. However, two patients were diagnosed late after a misdiagnosis: one was thought to have a contusion, and the other a patella dislocation. 

In Bradko et al., one patient was found to have an avulsion fracture and both patellar and tibial insertions on an MRI [16]. Previous radiographs showed a possible sleeve fracture and tibial tubercle fracture but missed this diagnosis. The other patient was treated for a sleeve avulsion fracture; the patellar tendon rupture was only identified during surgery [16].

In Yousef’s 2018 study [19] and Wood et al. [18], diagnosis of the patellar tendon rupture in all patients was only made during intraoperative surgery when the patient was being treated for a concomitant injury.

Patterns of Injury

Table 6 shows different rupture morphologies from four studies encompassing 11 patients that actually specified rupture location [15,16,18,19]. Where reported, the majority were distal tears of the patellar tendon. Only one case of a mid-substance tear was reported [15]. No paper mentioned whether ruptures were full thickness or a partial rupture.

**Table 6:** Types of rupture

Type of rupture/avulsion pattern	Number of patients
Distal patellar tendon avulsion from the proximal tibia / tibial tubercle	7
Proximal patellar tendon avulsion from the patella	1
Mid-substance patellar tendon rupture	1
Combined avulsion from tibial and patellar insertions	2

All five studies reported on associated injuries as summarised in Table 7. Of all reported associated injuries, tibial tubercle avulsion fractures comprised 70%. However, this is confounded by selection bias as three of the five studies specifically enrolled patients with tibial tubercle fractures. Soft tissue injuries were also described in some cases.

**Table 7:** Associated injuries

Concomitant injuries	Number of injuries
Tibial tubercle fracture / tibial tubercle avulsion fracture	21
Retinacular tear	4
Quadriceps muscle tear/strain at patella insertion	2
Contralateral tibial tubercle avulsion fracture	1
Sleeve avulsion fracture	1
Fat pad and anterior capsule avulsion from inferior patella	1

Various anatomical and medical associations were reported in patients sustaining patellar tendon ruptures. Patellar alta was identified in all five patients in Ali Yousef and Rosenfeld, and both patients in Bradko et al. [15,16]. Osgood-Schlatter disease was tested for in many studies and was reported in two patients [15,16]. One patient had osteogenesis imperfecta with DiGeorge syndrome, and another had Wolff-Parkinson-White syndrome with attention deficit disorder [15].

Management

All patients underwent operative management. The surgical techniques used across all studies are outlined in Table 8. This includes the treatment of all patellar tendon ruptures. Tibial avulsions were fixed in all cases when present in conjunction with tendon ruptures in the same repair. In some cases, patients underwent combined constructs. Techniques varied according to the site and pattern of rupture. 

**Table 8:** Management

Surgical technique	Number of patients
Suture anchor repair	12
Transosseous/bone tunnel suturing	8
Grasping suture to proximal tibia	6
Staple	6
Screw	6

Postoperative care generally involved a period of immobilisation followed by progressive rehabilitation. In Ali Yousef and Rosenfeld [15], one patient was immobilised using a long-leg cylindrical cast and extension block knee brace for six weeks; bracing was only recorded for one out of five patients. The mean duration of physiotherapy was 23.4 weeks [15]. In Bradko et al., one patient was placed in a knee immobiliser with crutches for four weeks, whilst the second was in a locked straight brace for a week before starting physiotherapy [16]. In Schultz et al., the mean time in a brace was 12.95 weeks [17]. In Wood et al., patients underwent immobilisation and protected weight bearing for four weeks, with physiotherapy starting at six weeks [18]. In Yousef’s 2018 study [19], two patients were placed in a knee immobiliser, with one later changing to a Bledsoe hinged knee. One patient was placed in a long-cast cylinder cast in full extension. One patient began physiotherapy after three weeks and continued for 21 weeks with sports-specific training.

Outcomes and Complications

In Ali Yousef and Rosenfeld, outcomes were assessed using active range of motion, manual quadriceps strength testing, presence of terminal extension lag, and a Knee Society Score [15]. Active range of motion ranged from 0-120° to 0-137° after a mean time of 18.4 weeks, with no terminal extension lag. Quadriceps strength was 5/5 in all patients. The mean time to return to sport was 22 weeks. The mean Knee Society Score was 91.8 with excellent outcomes in four patients and a good outcome in one [15].

At six weeks in Bradko et al., one patient showed radiographic healing, whilst the other was able to tolerate weight-bearing activity with a brace [16]. Both patients regained full range of motion by three months, and one had been permitted to return to sports activities. 

In Yousef’s 2018 study [19], active range of motion improved over follow-up. One patient achieved 0-130° by 12 weeks, another achieved 0-130° by 20 weeks, and the third achieved 0-120° by 12 weeks. 

In Schultz et al., the mean time to achieve full range of motion was 19.3 weeks [17]. The mean time to return to sports was 22.18 weeks. The study also showed that patients with both a tibial tubercle fracture and patellar tendon rupture required more time in a brace than patients with just a fracture [17]. 

Complications were reported in several studies. In Ali Yousef and Rosenfeld, one patient experienced a wound dehiscence requiring surgery to close the wound [15]. He later needed his FiberWire suture to be removed and suffered an ipsilateral quadriceps rupture requiring operative repair. Another patient required screw removal and had difficulties with activities involving jumping and decelerating [15]. In Yousef’s 2018 study [19], atrophy of the quadriceps muscle was noted in all three patients in the weeks after surgery. One patient also had limited patella mobility, peri-incisional numbness, and later had surgery to remove the screws due to palpable screw heads and mild pain.

Risk-of-Bias

Risk-of-bias assessment was performed using the JBI Critical Appraisal Checklist for Case Series for four papers, which were case series. The modified Newcastle-Ottawa scale was used for Schultz et al. [17], which is a retrospective observational study. The results can be found in Tables 9-10.

**Table 9:** JBI Critical Appraisal Checklist for Case Series N/A: not applicable

Author, year	Were there clear criteria for inclusion in the case series?	Was the condition measured in a standard, reliable way for all participants included in the case series?	Were valid methods used for identification of the condition for all participants included in the case series?	Did the case series have consecutive inclusion of participants?	Did the case series have complete inclusion of participants?	Was there clear reporting of the demographics of the participants in the study?	Was there clear reporting of clinical information of the participants?	Were the outcomes or follow up results clearly reported?	Was there clear reporting of the presenting site(s)/clinic(s) demographic information?	Was statistical analysis appropriate?	Overall appraisal
Ali Yousef and Rosenfeld, 2017 [15]	Yes	Yes	Yes	Unclear	Unclear	Yes	Yes	Yes	Yes	Yes	Include
Bradko et al., 2018 [16]	Unclear	Yes	Yes	Unclear	Unclear	Yes	Yes	Yes	Yes	N/A	Include
Wood et al., 2024 [18]	Unclear	Yes	Yes	Unclear	Unclear	Yes	Yes	Yes	Yes	N/A	Include
Yousef MA, 2018 [19]	Yes	Yes	Yes	Unclear	Unclear	Yes	Yes	Yes	Yes	N/A	Include

**Table 10:** Modified Newcastle-Ottawa scale ★: indicates satisfactory attainment of criteria -: indicates failure to meet criteria

Author, year	Representativeness	Selection of non-exposed cohort	Ascertainment of exposure	Outcome not present at start	Comparability	Outcome assessment	Was follow-up long enough	Adequate follow-up	Total score /9
Schultz et al., 2025 [17]	★	★	★	★	-	★	★	-	6

Discussion

This systematic review brings together all relevant evidence regarding presentation, diagnosis, management and outcomes of patellar tendon ruptures in children.

A key finding was that paediatric patellar tendon rupture often occurred as part of a wider extensor mechanism injury rather than as an isolated tendon rupture. Across all five studies, 21 of the 28 studied patients sustained tibial tubercle avulsion fracture alongside a patellar tendon rupture. This pattern differs from the more typical adult presentation of acute tendon rupture and likely reflects the relative vulnerability of the immature apophyseal and osteochondral structures in children and adolescents [16]. As a result, traumatic loading across the extension mechanism may produce avulsion-type injuries, sleeve injuries or combined bony and tendinous disruption. Therefore, examinations of patellar tendon ruptures in children should be considered as part of a wider assessment to include other aspects of the extensor mechanism.

Tibial tubercle fractures were a particularly important associated injury. Patellar tendon avulsion is the most common associated injury with tibial tubercle fractures, with rates of simultaneous injury between 4-15% [16]. The risk increases when adolescent patients are exposed to jumping or forceful quadriceps contractions [16,18]. Therefore, the diagnosis of a tibial tubercle fracture in children should warrant active investigation of the patellar tendon. This has practical implications for imaging and operative planning. Plain radiographs may identify the fracture but often fail to fully characterise the tendon injury, while MRI may be useful when clinical findings suggest more extensive soft tissue disruption. In addition, a high-riding patellar in plain X-rays was described by Ali Yousef and Rosenfeld as a hallmark diagnostic sign [15].

Another important finding is that most injuries occurred at the tendon insertions, with 9 of the 11 injuries where the location was mentioned being at the distal tibial insertion. Only one case of a midsubstance tear was recorded [15]. This likely reflects the biomechanics of the immature skeleton in children, in which the cartilaginous apophyses and osteotendinous junctions are weaker than the tendon itself [21].

The review also highlights the risk of missed diagnosis. In several included studies, the patellar tendon injury diagnosis was either delayed [15] or only revealed intraoperatively [16,18,19], since attention was directed towards other diagnoses. This suggests that the tendon component of an injury can often be overlooked when other injuries are present. Clinically, this is relevant because delayed recognition can affect recovery and rehabilitation. Children presenting with key signs including patellar alta, extensor lag or the inability to perform a straight leg raise should be assessed for patellar tendon disruption, even when other injuries have already been diagnosed. Nevertheless, intraoperative assessment is still important to rely on.

All patients were managed surgically. The included studies used a range of techniques, including transosseous sutures, suture anchors, staples and screws. Traditionally, transosseous tunnel repair has been regarded as the gold standard for patellar tendon ruptures, particularly in adult practice [8]. More recently, suture anchor repair has become increasingly common. Biomechanical literature has suggested that it can lead to reduced gap formation compared to transosseous repair [22]. Transosseous repair is also being less opted for due to re-rupture rates [17]. This trend was also reflected in the paediatric literature, particularly for distal avulsion injuries and when combined with a tibial tubercle injury [16,17]. However, current evidence is still insufficient to determine whether suture anchors are superior to transosseous repair in children. At present, technique is guided largely by injury location, associated fracture pattern and surgeon preference.

Post-operative rehabilitation was inconsistently reported amongst the studies. Most described a period of immobilisation or bracing in knee extension in order to relieve tension off the tendon, which was followed by physiotherapy. However, they varied in their duration, weight-bearing status and the time to return to sport. This limits the ability to recommend a specific rehabilitation pathway. It is likely that injuries also involving bone fractures require a more cautious approach, as Schultz et al. [17] showed that time in a brace was significantly higher in patients with combined injuries than those with a tibial tubercle fracture alone. Future studies should report rehabilitation protocols in greater detail, including bracing duration, range of motion progression, weight-bearing restrictions and criteria to return to sport.

It should be acknowledged that this review has several limitations in the level of evidence. Four of the five studies were case series, and one was a retrospective observational study. There was also substantial heterogeneity with regard to many of the aims, including diagnosis, rehabilitation and outcome reporting.

Future research should focus on larger multi-centre studies or prospective registries due to the rarity of the condition. More consistent reporting of diagnostic pathways, rehabilitation protocols and follow-up of patients to determine long term outcomes is required. Studies should use standardised definitions to distinguish between isolated tendon ruptures from combined injuries.

## Conclusions

Paediatric patellar tendon rupture is a rare but important injury that should be considered in children and adolescents presenting with acute knee trauma and extensor mechanism dysfunction. The available evidence suggested that these injuries often occur as avulsion-type combined injuries, particularly with tibial tubercle fractures. Surgical repair generally led to good reported outcomes. However, this finding is based on limited heterogenous evidence. Larger multicentre studies with standardised reporting are needed to guide future management. 
